# Testicular trauma resulting in shock and systemic inflammatory response syndrome: a case report

**DOI:** 10.1186/1757-1626-1-4

**Published:** 2008-05-12

**Authors:** Kingsley C Okonkwo, Kristin G Wong, Cheng T Cho, Lisa Gilmer

**Affiliations:** 1 University of Kansas, Department of Pediatrics, Kansas City, Kansas, USA

## Abstract

**Introduction:**

Acute painful scrotum in children may be associated with torsion of the testis, hematocele, epididymitis and direct testicular injury with hematoma formation. More frequently, however, acute scrotum occurs without a precipitating factor. While most traumatic testicular injuries resolve with conservative management, many require surgical exploration and some are life-threatening.

**Case presentation:**

A 13-year-old boy with a history of testicular trauma presented with severe scrotal swelling and shock. This case study examines the presentation and possible role of cytokines in the development of systemic inflammatory response syndrome in a child with acute traumatic epididymitis.

**Conclusion:**

Post-traumatic epididymitis presenting as shock in boys is rarely reported. We advocate early recognition of the chain of events leading to clinical presentation of shock and prompt treatment to preserve testicular viability.

## Introduction

Acute scrotal swelling in children is often associated with testicular torsion, testicular rupture, hernia, epididymitis and direct testicular trauma [1], although acute scrotum most frequently occurs without a precipitating factor. In a previous study testicular torsion, torsion of testicular appendages, and epididymitis were found to represent 94% of all final diagnoses in boys under the age of 17 years who were hospitalized for acute scrotal pain and swelling [2]. Many of these conditions require conservative treatment while others require surgical exploration when accurate diagnosis cannot be made from physical examination and diagnostic imaging. A few cases result in systemic inflammatory response syndrome (SIRS) requiring life-saving therapy. SIRS can be seen with or without evidence of infection in cases of trauma. The purpose of this paper is to report an unusual case of testicular trauma resulting in life threatening SIRS.

## Case presentation

A previously healthy 13-year-old Caucasian boy presented with testicular trauma to a community hospital and was transferred to the University of Kansas Hospital (KUHA) pediatric intensive care unit (PICU) for management of hypotension and swollen painful testicles. Five days prior he had been kicked in the groin during a football game and presented to his primary care physician with scrotal pain and swelling of his scrotum. Initial treatment consisted of rest and watchful waiting.

Two days post-trauma he developed fever and nausea while at home and was taken to the emergency department of an outside facility. On examination the patient's scrotum was extremely swollen and tender, and his testes were firm to the touch. Urinalysis showed an elevated white blood cell count (WBC) and red blood cell count, and was positive for ketones, hemoglobin, leuko-esterase, and bacteria; however, gram stain and urine culture revealed no organisms. Blood culture also showed no growth of organisms. Ultrasound at this time showed findings suggestive of bilateral epididymitis with no evidence of testicular torsion or mass. The patient was discharged in stable condition on hydrocodone for pain and doxycycline for epididymitis with instructions to follow up with urology if symptoms worsened.

Four days post-trauma, the patient presented to the urologist complaining of blood in his urine, lightheadedness, nausea, vomiting, and shortness of breath secondary to severe scrotal pain. On physical examination his scrotum was enlarged, warm, tender, and firm. He was admitted with a WBC of 38,600/mm^3 ^with a left shift, and normal blood chemistry. The patient also had a temperature of 38.8°C, C-reactive protein (CRP) of 20 mg/dl, and an erythrocyte sedimentation rate of 80 mm/hour. Urinalysis was unchanged from the previous results. Ultrasound showed findings of bilateral epididymitis and echogenic material surrounding both testes, which was slightly more prominent in volume compared with the study done 2 days prior. Abdominal and pelvic computed tomography scan found shotty inguinal and periaortic nodes, as well as, mesenteric adenopathy. No discrete abscess or diffuse abnormal inflammation was seen. Doxycycline was discontinued and IV ceftriaxone was started empirically, although no microbiological organism was cultured from the patient's blood or urine. He was later switched to vancomycin and meropenem for broader antimicrobial coverage.

**Figure 1:**
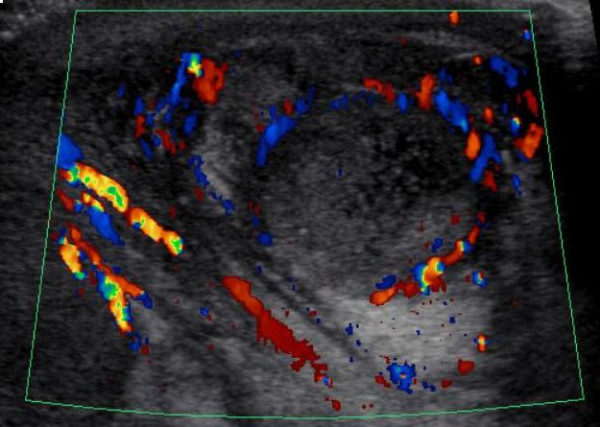
**Ultrasound scan at case presentation**. Doppler ultrasound scan image showing focal heterogeneity with decreased vascularity in the left upper testicle consistent with a small laceration, subscapular hematoma and intraparenchymal hematoma.

On the fifth day post-trauma the patient remained febrile with progressing scrotal swelling and pain. The patient subsequently developed hypotension with a blood pressure drop as low as 87/22 mmHg, at which time; he was started on a dopamine drip at 13 mcg/kg/minute. An electrocardiogram performed at the time showed normal sinus rhythm. Once stabilized, he was transferred to KUHA's PICU for further management. Upon arrival his temperature was 39°C and heart rate was 108 beats per minute. The patient's blood pressure dropped again to 84/20 mmHg but was stabilized with dopamine and fluids. The patient's scrotum was swollen, erythematous, and his left testicle was more tender than his right. He had a WBC of 34,600/mm^3^, and CRP of 33 mg/dl. Doppler ultrasound scan of the scrotum (Fig. 1) showed focal heterogeneity with decreased vascularity in the left upper testicle consistent with a small laceration, subscapular hematoma and intraparenchymal hematoma.

Blood and urine cultures at presentation to the PICU were negative for organisms. Ceftriaxone was restarted as his antibiotic regimen. The patient's WBC and CRP gradually declined while his scrotal sonograms showed little change during hospitalization. The patient's clinical condition gradually improved with continued antimicrobial therapy, scrotal elevation, ice and rest. The urological consult did not find evidence of abscesses or torsion and therefore did not recommend surgical intervention.

By day 10, the patient's scrotal exam had dramatically improved, his WBC had normalized, CRP was 4.38 mg/dl and he had been afebrile for 4 days. His blood and urine cultures at initial presentation to the community hospital and at our facility were all negative. He was discharged on Levaquin 750 mg for 11 days to complete 21 days of therapy.

**Figure 2:**
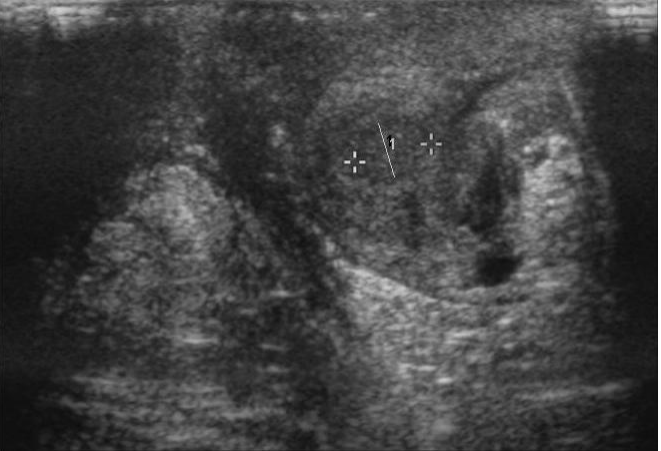
**Ultrasound scan 3 months later**. Ultrasound scan image showing decrease in size of the left testicle with increased nodularity in the previously injured region of the left testicle consistent with post-inflammatory scarring.

At the 6-week follow up appointment the patient had significant improvement in scrotal appearance and swelling with some continued mild edema of the scrotal subcutaneous tissue and firm testes. His WBC was 9600/mm^3 ^with a normal differential and on ultrasound there was interval decrease in bilateral testicular size with persistent diffuse moderate heterogeneity. These findings were consistent with resolving swelling and edema from post-traumatic orchitis and/or intraparenchymal hematoma and/or injury. Three months after the initial testicular trauma, the patient's testes were smaller and softer on examination and findings on ultrasound (Fig. 2) were consistent with post-inflammatory scarring.

## Discussion

Traumatic injuries to the genitalia are relatively uncommon and rarely result in life-threatening circumstances [1]. In cases of testicular injury, blunt scrotal trauma is responsible for 75% of the reported cases; of these, most result from sports injuries, vehicle accidents and assault [1]. Regardless of the severity and mechanism of injury, immediate assessment of the genitalia is warranted to rule out testicular torsion or rupture. Other diagnoses of concern include urethral injuries, hematomas and epididymitis. One study looked at acute scrotal pain in boys under 17 years of age and found that testicular torsion, torsion of a testicular appendage, and epididymitis accounted for 94% of the diagnoses [2]. While most cases of epididymitis are the result of bacterial infection, some may present 2 to 3 days after trauma due to forceful compression of the testis against the pubic bones [3].

Once ultrasonography rules out the need for surgical intervention, scrotal injuries can be managed conservatively with ice, elevation, and analgesics [1]. In one study of external genital injuries during childhood, 66.5% involved the scrotum or its contents and 73.1% were managed with conservative treatment [4]. In our patient surgical intervention was never warranted because of the absence of torsion, significant hematoma, testis rupture, or abscess. It is generally believed that scrotal injuries are not life-threatening and are more concerning for their long-term complications [5]. In this case, despite conservative management and empiric antibiotics, our patient's condition worsened over the first days and progressed to SIRS requiring life-saving intervention.

SIRS, as defined by the American College of Chest Physicians and Society of Critical Care Medicine, requires that two or more of the following conditions are present: temperature >38°C or <36°C; heart rate >90 beats/minute; respiratory rate >20 breaths/minute or P_a_CO_2 _<32 torr; WBC >12,000 cells/mm^3^, <4000 cells/mm^3^, or >10% band forms. In the presence of proven infection, the term 'SIRS' becomes 'sepsis'. The difference in terminology arises from the observation that trauma patients with or without infections have similar outcomes, suggesting the body's overall response is more predictive of clinical course than the particular organism or mechanism of injury [6]. Noninfectious causes of SIRS include acute pancreatitis, burns, trauma, or major elective surgery [7].

Upon initial presentation our patient met four of the SIRS criteria, and while no culture evidence of bacteremia or abscess was found, it was difficult to distinguish between culture-negative SIRS versus noninfectious SIRS. Presently there is no specific or sensitive test to distinguish between the two diagnoses due to the expansive and overlapping systemic responses in infection and trauma [7], making medical management difficult and potentially delaying recovery. Our patient never showed symptoms of a urinary tract infection, did not have risk factors for sexually transmitted infections, and did not develop signs of an abscess, giving us no reason to suspect sepsis as opposed to SIRS.

Our patient's history of trauma without other underlying medical conditions led to the belief that his diagnosis of SIRS was the result of a cytokine storm released from direct trauma to the scrotum. Although the testis is an immune-privileged site, it is not completely protected from local and systemic activators of inflammation [8]. The theory behind cytokine storms lies in the ability of the immune system to control the response to injuries or infection. A cytokine storm is a potentially fatal immune reaction consisting of a positive feedback loop between cytokines and immune cells [9]. Cytokines, such as tumor necrosis factor-α, interleukin-1, and toll-like receptors, are highly involved in the activation and progression of the inflammatory response system [9]. Additionally, these same cytokines have been studied in the role of Leydig cell steroidogenesis, and thus are not only present in macrophages, T cells, and other circulating immune cells, but are also in Sertoli cells and developing germ cells of the testes [8]. Consequently, severe trauma to the scrotum and its contents can lead to a release of pro-inflammatory mediators with the potential to become overwhelming and systemic in nature. Testing for cytokines is not routinely performed and our patient was not tested. In view of the lack of laboratory confirmation of cytokine responses in our patient, the interpretation of cytokine storm cannot be made with certainty.

Current studies of immune regulation include potential therapies for combating SIRS before patients suffer severe complications. Mortality increases with increasing numbers of SIRS criteria present in a patient; in one study mortality doubled in patients with SIRS to 6% compared to those without, and increased to 17% in those matching four criteria [6]. Thus far, transcription factors such as nuclear factor-κB and activating protein have been targeted as possible treatment options for these patients [9]. However, these options are not currently available for therapeutic use. It may still be unwise to suppress the immune system, even without evidence of infection; thus we propose that the standard of care should include empiric antibiotics without immune suppression.

## Conclusion

As the treatment for SIRS continues to be studied, it is important to recognize its signs and symptoms and to remain aware of its occurrence in seemingly unlikely cases. In our case, the patient initially presented with a minor scrotal injury; swelling and pain without ecchymosis were the only symptoms. Then, without evidence of infection, he gradually progressed to a life-threatening condition, unresponsive to empiric antibiotics and standard fluid resuscitation. Now recovered, the patient continues to be monitored for any long-term consequences of his injury. Initially patients with acute scrotal pain are told to look for signs of injury and infection, but they should also be aware of the signs of sepsis and SIRS. Although rare, death is no longer beyond the realm of consequences from testicular injury and physicians must be prepared to deliver appropriate care.

## Abbreviations

CRP: C-reactive protein; KUHA: University of Kansas Hospital; PICU: pediatric intensive care unit; SIRS: systemic inflammatory response syndrome; WBC: white blood cell count.

## Competing interests

The authors declare that they have no competing interests.

## Authors' contributions

KCO, KGW, CTC and LG conceived the study and participated in its design, coordination and helped to draft the manuscript. All authors read and approved the final manuscript.

## Consent

Written informed consent was obtained from the patient's parents for publication of this case report and any accompanying images. A copy of the written consent is available for review by the Editor-in-Chief of this journal.
